# High density lipoproteins and oxidative stress in breast cancer

**DOI:** 10.1186/s12944-021-01562-1

**Published:** 2021-10-25

**Authors:** Gabriele Mazzuferi, Tiziana Bacchetti, Md Obaidul Islam, Gianna Ferretti

**Affiliations:** 1 Department of Clinical Sciences, Section of Biochemistry, Biology and Physics, Polytechnic University of Marche, Ancona, Italy; 2 Department of Life and Environmental Sciences, Polytechnic University of Marche, Ancona, Italy

**Keywords:** Lipoprotein, High-density lipoprotein, Oxidative stress, Cholesterol, Breast cancer

## Abstract

Breast cancer is one of the main leading causes of women death. In recent years, attention has been focused on the role of lipoproteins, alterations of cholesterol metabolism and oxidative stress in the molecular mechanism of breast cancer. A role for high density lipoproteins (HDL) has been proposed, in fact, in addition to the role of reverse cholesterol transport (RCT), HDL exert antioxidant and anti-inflammatory properties, modulate intracellular cholesterol homeostasis, signal transduction and proliferation. Low levels of HDL-Cholesterol (HDL-C) have been demonstrated in patients affected by breast cancer and it has been suggested that low levels of HDL-C could represent a risk factor of breast cancer. Contrasting results have been observed by other authors. Recent studies have demonstrated alterations of the activity of some enzymes associated to HDL surface such as Paraoxonase (PON1), Lecithin-Cholesterol Acyltransferase (LCAT) and Phospholipase A2 (PLA2). Higher levels of markers of lipid peroxidation in plasma or serum of patients have also been observed and suggest dysfunctional HDL in breast cancer patients. The review summarizes results on levels of markers of oxidative stress of plasma lipids and on alterations of enzymes associated to HDL in patients affected by breast cancer. The effects of normal and dysfunctional HDL on human breast cancer cells and molecular mechanisms potentially involved will be also reviewed.

## Introduction

Breast cancer (BC) is one of the main leading cause of women death [1]. Hormonal, lifestyle and environmental factors increase risk of breast cancer. Moreover obesity and diabetes contribute to the increased risk [2, 3]. Previous studies have demonstrated alterations of levels of plasma lipoprotein involved in cholesterol transport and higher markers of oxidative stress in obese and diabetic patients [4, 5]. Therefore, in recent years, attention has been focused on the role of plasma lipoproteins, alterations of cholesterol metabolism and oxidative stress in the molecular mechanisms of breast cancer [6–10]. A role for high density lipoproteins (HDL) has been hypothesized. In fact, in addition to the reverse cholesterol transport, HDL exert pleiotropic roles such as antioxidant and anti-inflammatory properties [11, 12]. Alterations of HDL properties and functions and reverse cholesterol transport have been widely studied in human diseases associated with oxidative stress [11, 12]. Moreover, a relationship between low serum HDL levels and risk of cardiovascular disease has been demonstrated [13]. Plasma levels of cholesterol associated to HDL (HDL-C) lower than 35 mg/dl, represent a risk factor for the development of atherosclerotic disease [13]. Modifications of serum total cholesterol and cholesterol associated to HDL are also described in patients affected by different tumours [14]. Several studies have demonstrated that also patients with breast cancer have abnormal levels of HDL-C [15–25]. However, several discrepancies are described. A positive correlation between high serum level of HDL-C and breast cancer risk has been observed by some authors [15] and two recent Mendelian randomization analyses have demonstrated that genetically predicted levels of increased HDL-C were associated with increased breast cancer risk [18–20]. Other studies have demonstrated a negative correlation between HDL-C and breast cancer. An inverse association between HDL-C and breast cancer risk has been observed by Touvier et al. in premenopausal women [21]. However only a modest association between low HDL-C and increased incidence of breast cancer among women who were pre-menopausal has been observed by Kucharska et al. [22]. A higher risk of death in triple negative breast cancer (TNBC) is described in patients with low serum levels of HDL-C [23]. Even Lofterød et al. [24] have shown that the ratio HDL-cholesterol/total-cholesterol ratio may independently provide useful information on the prognostic outcome among TNBC patients. Levels of HDL-C have been confirmed as a protective factor for overall survival in cancer patients by the meta-analysis of Zou et al. [25]. Differences in study population, menopause state of patients, study design (inclusion and exclusion criteria), state of the disease, ethnic factors could explain the discrepancies.

Although contrasting results have been reported on the relationship between breast cancer risk and HDL-C, an increasing interest is devoted to the role of HDL and molecular mechanisms of breast cancer development. In fact, a modulatory role of HDL on intracellular cholesterol content and homeostasis has been described. Alterations of cholesterol levels are described in animal models of breast cancer [9]. It is well known that cholesterol exerts several physio-pathological roles. Higher levels of cholesterol are considered essential for cancer cell proliferation and tumor progression and mitochondrial cholesterol levels induce resistance to apoptotic signals [26, 27]. Cholesterol exerts also a modulatory role of cell membrane physico-chemical properties (order and fluidity) [28]. Lipid rafts and cholesterol-rich domains contain several signaling receptors such as epidermal growth factor receptor (EGFR) [29]*.* The physio-pathological roles of cholesterol are also related to the high sensibility to lipid peroxidation. Oxysterols (OS), oxidized derivatives of cholesterol, are bioactive lipids involved in regulation of several cellular mechanisms [30]. OS may enter the circulation as products of lipid peroxidation on cholesterol-containing food, may be generated by enzymatic reactions or by autoxidation processes in the presence of free reactive oxygen species [30, 31]. Both cholesterol hydroperoxide and cholesterol epoxide can be evaluated in plasma samples of patients affected by dysmetabolic diseases associated with oxidative stress [32]. Circulating OS are described also in BC patients [33, 34]. During BC development, alterations of cholesterol and oxysterols metabolism are described. Conflicting results have been reported on the roles of 27-hydroxycholesterol (27-HC), a primary metabolite of cholesterol. Studies in experimental animal models and cells in culture have demonstrated that 27-HC promotes breast cancer cell growth and metastasis [35, 36]. In contrast, recent clinical reports showed that higher circulating levels of 27-HC were associated with lower risk of breast cancer in postmenopausal women [34]. Moreover limited associations between levels of 27-HC and breast cancer characteristics have been observed by Le Cornet et al. [37]. The potential BC promoter properties of 5,6-epoxycholesterol (5,6-EC) is also debated. 5,6-EC derive from autoxidation and photooxidation of cholesterol. 5,6-EC has been shown to be differentially metabolized in breast cancers compared to normal breast tissue and is metabolized into the 6-oxo-cholestan-diol (OCDO) [38]. Both enzymes the cholesterol-5,6-epoxide hydrolase (ChEH) and the 11beta-hydroxysteroid dehydrogenase type 2 (HSD2) involved in OCDO biosynthesis are upregulated in BC tissue; therefore it has been suggested that 5,6-EC could favor BC development. Studies in vitro have demonstrated that OCDO, recently named Oncosterone, behaves as a tumor promoter on estrogen receptor ER+ cells and in TNBC via the activation of the glucocorticoid receptor [38–40]. The alterations of OS metabolic pathways in BC and their potential opportunities for BC treatment have been recently reviewed [41].

**Fig. 1:**
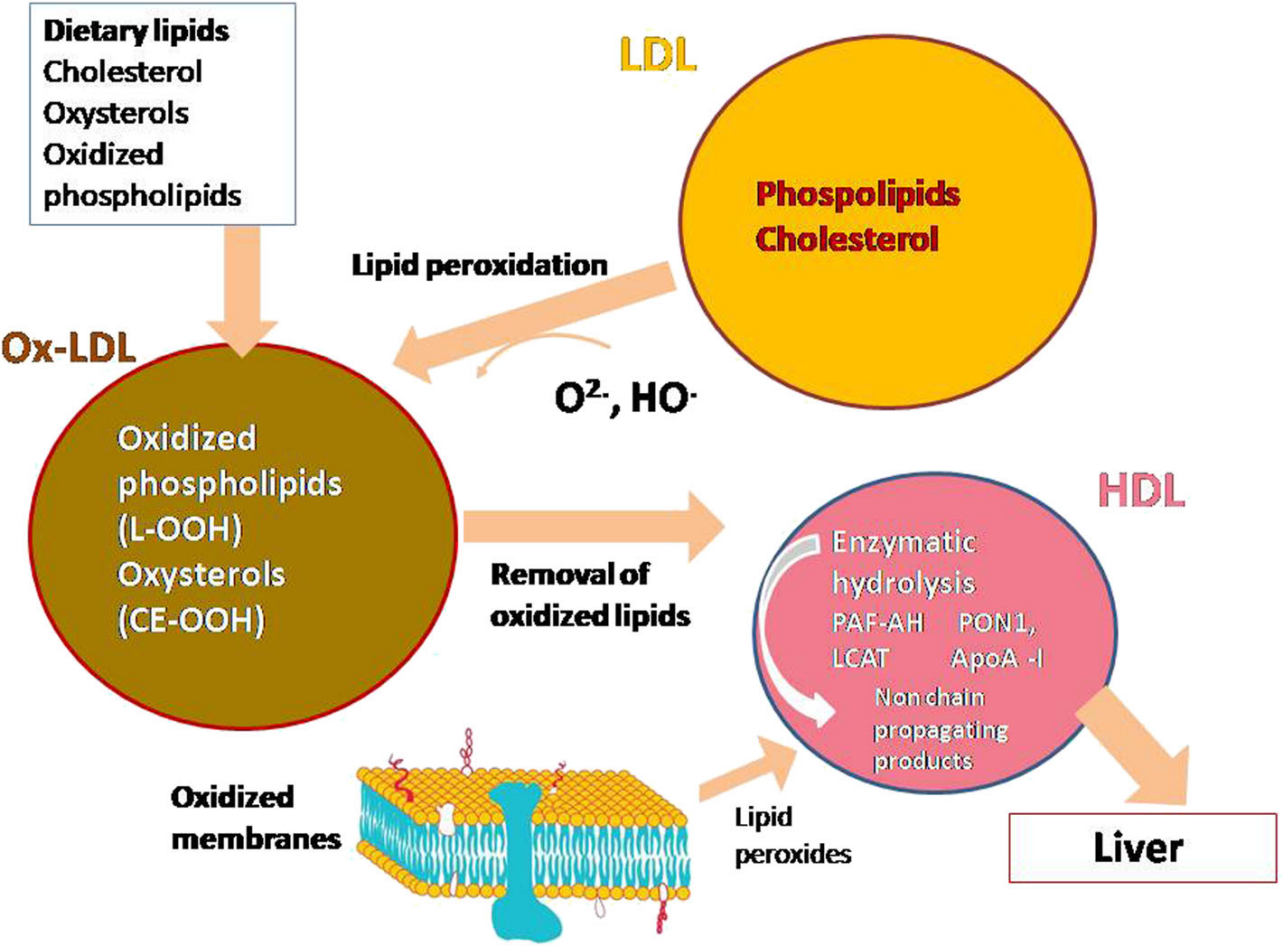
The role exerted by high density lipoprotein (HDL) in the metabolism of lipid hydroperoxides and oxysterols. Lipid hydroperoxides or oxysterols can be formed on LDL and migrate to the surface as a result of their greater hydrophilicity, facilitating their transfer to HDL

The interest to the study of HDL in the mechanisms of human diseases associated to oxidative stress, is also related to their antioxidant properties. The ability of HDL to inhibit lipid peroxidation of LDL and biological membranes is widely studied as a protective mechanism against development of chronic diseases [11, 12, 42–44]. However it has also to be stressed that HDL are considered a vehicle for circulating products of lipid peroxidation of phospholipids [45] and efflux of some OS to HDL has been described [46]. For instance HDL transport oxysterols including cholesterol esterified hydroperoxides (CEOOHs) and 27-hydroxycholesterol (27-HC) [46]. It has been also demonstrated that HDL are able to remove an excess of OS (7-ketocholesterol and 7α/β-hydroxycholesterol and 7α-,27-hydroxycholesterol) from THP1 macrophages and from the surface of oxidized low density lipoproteins (oxLDL) [46, 47]. OS uptake to HDL allows to decrease intracellular levels of cytotoxic OS. Furthermore, HDL is the main carrier of molecules formed upon oxidation of arachidonic acid such as plasma F_2_-isoprostanes (F_2_-IsoPs) [48]. The function of HDL to accept and transport oxidized lipids may ensure efficient elimination of lipid peroxidation products from the circulation through the liver [49] (Fig. 1).

A key role in the antioxidant properties of HDL is exerted by the enzyme paraoxonase-1 (PON1) associated to HDL surface. PON1 hydrolyses oxidized lipids and protects LDL and biological membranes from lipid peroxidation [42–44]. Therefore, subjects with lower PON1 activity are more susceptible to oxidative damage when compared with subjects with higher PON1 activity. PON1 is also involved in the detoxification of carcinogenic, lipid-soluble free radicals produced after lipid peroxidation [50, 51]. Other enzymes appear involved in biological properties and antioxidant role of HDL such as platelet activating factor acetyl hydrolase (PAF-AH), phospholipase A2 (PLA2) and lecithin cholesterol acyltransferase (LCAT) [52, 53]. The enzyme PAF-AH associated to HDL or LDL behaves as calcium-independent phospholipase A_2._ and degrades PAF, a potent inflammatory mediator [52, 53]. Moreover PAF-AH in HDL is able to remove phospholipid core aldehydes because of its phospholipase A_2_ activity. LCAT plays also a role in HDL maturation [54]. Alterations of the activity of enzymes associated to HDL surface have been demonstrated in serum and plasma of BC patients [55, 56]. Literature data report that oxidative stress and lipid peroxidation could be involved in the pathogenesis of BC [10, 57]. The potential roles of plasma lipoproteins in breast cancer have been recently reviewed [6–8]. However, the relationship between breast cancer, HDL and oxidative stress has not been reviewed. In detail, our review is focused on markers of plasma lipoprotein peroxidation and on alterations of HDL-associated enzymes and apolipoproteins in patients affected by breast cancer. The effects of normal and dysfunctional HDL on human breast cancer cells and the molecular mechanisms potentially involved will be also reviewed.

### Circulating markers of oxidative stress of breast cancer patients

The relationship between lipid peroxidation of plasma lipoproteins and BC is supported by the higher levels of markers of lipid peroxidation in plasma or serum of BC patients as summarized in Table 1. Elevated serum levels of lipid hydroperoxides and oxLDL have been observed in BC patients [62] (Table 1). An increase of the ex-vivo susceptibility of serum lipids to oxidation and anti-oxLDL autoantibodies have also been observed [61]. Furthermore, serum levels of oxLDL were associated with higher BC risk [61]. Balci et al. [55] and Samra et al. [56] have confirmed that breast cancer is associated with lipid peroxidation of plasma lipids. Higher levels of lipid hydroperoxides (LOOH) and malondialdehyde (MDA) have been demonstrated in BC patients by other authors [60, 63–66] (Table 1).

**Table 1:** Levels of markers of oxidative stress in serum and plasma of controls and BC patients

Markers oxidative stress	Control	BC patients	Reference
Serum lipid hydroperoxides (μmol/L)	1.08 ± 0.24	1.97 ± 0.50	[58]
Plasma lipid hydroperoxides (nmol/mL)	43.13 ± 9.12	58.23 ± 07.12	[59]
Serum malondialdehyde (nmol/mL)	1.92 ± 0.12	3.77 ± 0.25	[60]
Serum malondialdehyde (μmol/L)	2.72 ± 0.22	3.64 ± 0.25	[61]
TBARS (μmol/L)	2.24 ± 0.83	2.62 ± 0.96	[56]
OxLDL (U/L)	78.8 ± 56.1	288.3 ± 262.3	[61]
Ex-vivo susceptibility of serum lipids to oxidation (OD/sec)	9.89*10^−5^ ± 3.36*10^− 5^	13.33*10^− 5^ ± 1.35*10^− 5^	
Anti-oxLDL autoantibodies (U/L)	466.1 ± 495.7	737.9 ± 388.8	

Even oxysterol levels are detected in the circulation of breast cancer patients. As aforementioned, recent studies have demonstrated the clinical relevance of circulating levels of oxysterols in BC patients [33, 34]. In particular, levels of cholestane-3β,5α,6β-triol (CT), may have prognostic roles in patients with luminal subtype breast cancer [38, 39].

A decrease of the serum levels of total antioxidant capacity in non-metastatic and metastatic BC patients than the healthy controls has also been observed [65, 66]. All these data confirm that oxidative stress of plasma lipoproteins occurs in BC. As aforementioned, plasma HDL behave as carriers of lipid peroxidation products and play a role in their detoxification [45, 49, 58, 59].

It has to be stressed that dietary oxidized fatty acids and oxisterols such as 5,6-epoxycholesterols (5,6-EC) and CT are absorbed by the small intestine and incorporated into chylomicrons (CM) [31]. Athough oxisterols are present in very low concentrations in plasma with respect to cholesterol (10^4^- to 10^6^-fold times less), they circulate assembled on VLDL,LDL,HDL and increase lipoprotein susceptibility to oxidation. Their concentrations can increase with circulating cholesterol concentration and diet [31]. Oxidized sterols in the diet have been mainly studied in the mechanisms of atherosclerosis [59]. The role of processed foods containing oxysterols or precursors of OCDO deserves of further studies. In fact processed meats and ultra-processed foods with high amounts of cholesterol, have been associated with a significant increase in breast cancer risk [67, 68].

As far as concerns the physio-pathological role of the increased levels of lipid peroxidation products, it is well known that they play a role in oncogenesis [69]. Reaction between MDA and functional groups of a variety of cellular biomolecules can cause structural and functional damage [69]. The relationship between oxidative stress and BC is also supported by the increase 8-F2 isoprostanes in BC patients [70]. The increased levels of LOOH, MDA,OS and 8-F2 isoprostanes in plasma and/or serum of BC patients, suggest that HDL could behave as dysfunctional lipoproteins. Literature data confirm that oxidized HDL exert alterations of their properties with a lower protective affect against oxidative stress and a lower efflux of cholesterol or oxysterol (Fig. 1).

### HDL associated apolipoproteins and enzymes in breast cancer patients

As aforementioned, HDL exert several biological roles which involve apolipoproteins, enzymes (PON1, PAF-AH, LCAT) and bioactive molecules (Table 2). The antioxidant effects of HDL have been previously studied in vitro and in vivo [42–44]. Intravenous injection of HDL induces a decrease in the levels of products of peroxidation in animal models [71]. We summarize here literature data on alterations of HDL-associated apoproteins, enzymes and bioactive molecules in breast cancer patients (Table 3).

**Table 2:** Bioactive components associated to HDL

Molecule	Roles
ApoA-I	Structural role, lipid transport, antioxidant properties, immune response, haemostasis
Lecithin-Cholesterol Acyltransferase (LCAT)	Role in HDL maturation, convertion of free cholesterol into cholesteryl ester, antioxidant properties
Paraoxonase (PON1)	Antioxidant and anti-inflammatory properties of HDL, modulation of cholesterol efflux
Phospholipase A2 (PLA2) Platelet-activating factor-acetylhydrolase (PAF-AH)	Hydrolysis of acetyl ester at the sn-2 position of PAF. Antioxidant properties of HDL
miRNA	Regulation of gene expression
Serum amyloid A (SAA)	Acute-phase protein
Sphingosine-1-Phosphate (S1P)	Signaling molecule that regulate cell growth, survival and differentiation, suppression of inflammation

**Table 3:** Levels of activities and/or proteins associated to HDL of controls and BC patients

	Control	BC patients	Reference
Serum paraoxonase (PON, U/mL)	124.89 ± 21.70	42.99 ± 7.98	[55]
	158.39 ± 23.04	96.44 ± 21	[92]
Serum arylesterase (ARE) (U/mL)	98.55 ± 18.82	54.51 ± 11.57	[55]
	239.33 ± 32.98	159.75 ± 15.75	[92]
Plasma paraoxonase (PON)(U/mL)	95.00 ± 30.38	58.50 ± 1.67	[93]
Plasma arylesterase (ARE) (U/mL)	52.10 ± 11.98	65.00 ± 28.29	
PON levels (μg/mL)	80.62 ± 02.56	58.50 ± 1.67	
PON1 activity (U/L)	274.3 (156.8–565.9)	154.9 (99.9–260.2)	[92]
PON1 concentration (mg/L)	97.3 (43.2–285.3)	91.7 (30.6–223.3)	
Plasma LCAT (pg/ml)	5.98 ± 0.88	5.12 ± 0.58	[94]
Plasma PLA2 activity	1600 ± 100	1800 ± 100	[95]

#### Apolipoproteins

HDL are a heterogeneous class of lipoproteins. Like other mature lipoproteins, HDL contain neutral lipids (cholesteryl esters, triglycerides) in their core. At the surface there are glycerophospholipids, sphingolipids and free cholesterol. ApoA-I and apoA-II are the main apolipoproteins [72, 73]. ApoA-I is about 70% of the protein content associated with HDL. Several other proteins contribute to diverse functions including lipid transport, immune response, antioxidant properties, hemostasis. Alterations of apolipoprotein and lipid composition have been observed in patients affected by chronic diseases [74, 75]. ApoA-I exerts a structural role and provides stability to HDL particles in various stages of maturation. In addition ApoA-I appears involved in some pleiotropic roles of HDL and in cholesterol efflux from peripheral tissues. ApoA-I is also essential for the stability of the enzymes PON1 and LCAT. Moreover, an enzyme-independent antioxidant activity of apoA-I and apoA-II has been proposed by Garner et al. [76]. Phospholipid hydroperoxides and cholesteryl ester hydroperoxides are reduced to their correspondent hydroxides. The reduction of lipid hydroperoxides is associated with the formation of oxidized forms of methionine residues of apoA-I and apoA-II [77, 78]. Therefore, HDL converts potentially reactive species to relatively inert species as summarized in Fig. 1). Previous studies revealed that apolipoproteins are likely involved in mechanisms involved in cancer development [79]. Contrasting results have shown on levels and roles of apoA-I in BC. Some studies have demonstrated a greater association between apoA-I values and the development of breast cancer than for HDL-C values in human patients [80]. Studies in vivo in mammary tumour virus-polyoma middle T-antigen transgenic (PyMT) mice as a model of inherited breast cancer, have demonstrated that overexpression of human apoA-I induced an increase in the HDL-C level, but it did not affect tumour onset and growth [81]. On the contrary apoA-I mimetic peptide (D-4F), increased tumour latency and exerted a inhibitory effect against the development of tumours [81]. Furthermore, the D-4F treatment was associated with a significant decrease of the plasma levels of oxLDL and inhibited oxLDL-mediated proliferative response in human breast adenocarcinoma MCF-7 cells [81].

In contrast, other studies suggested a positive association between levels of apoA-I and BC [82–84]. Therefore, the physio-pathological relevance of the modifications of apoA-I levels merits of further studies. The apolipoprotein is transcribed by apoA-I/C3/A5 gene cluster, which modulates HDL synthesis and activity of the enzyme lipoprotein lipase [85]. Studies of apoA-I single nucleotide polymorphisms (SNPs) in breast carcinomas suggest that genetic variations of apoA-I, may represent a marker for the increased risk of BC [86]. A study on Taiwanese breast cancer patients [87] has verified the direct correlation between apoA-I rs670 minor allele and the chances of survival of the analyzed subjects. Patients carrying both minor alleles had the worst survival in lymph node-negative patients.

#### Paraoxonase 1 (PON1)

Among enzymes associated with HDL antioxidant functions, PON1 has been widely investigated [42–44, 88, 89]. The protective effect of HDL-bound PON1 against lipid peroxidation of membranes, LDL and HDL has been widely demonstrated. PON1 behaves as a lactonase and is a calcium-dependent enzyme [90]. Aviram et al. [91] demonstrated that PON1 hydrolyses either phospholipid peroxides or cholesteryl linoleate hydroperoxides in HDL oxidized in vitro. Furthermore,purified PON1 is able to hydrolyze hydrogen peroxide (H_2_O_2_). Alterations of PON1 activity have been widely demonstrated in several human chronic diseases and in cancer [34, 92]. Lower activities of paraoxonase (PON) and aryl esterase (ARE) have been shown also in BC patients (Table 3) [44, 45, 93, 96]. Lower levels of PON1 protein have also been described [96]. As far as concerns the physiological relevance of the observed alterations, Okuturlar et al. [93] have shown that serum PON and ARE levels were lower in patients who needed neoadjuvant chemotherapy than in patients who did not need the therapy. In addition, Bobin-Dubigeon et al. [96] found that PON1 was an independent factor of early death in BC recurrence. Previous studies have shown that PON1 and of serum amyloid A (SAA) are carried in the circulation bound to HDL. In case of inflammation their levels tend to modify in opposite directions [97]. It has been suggested that the evaluation of SAA and ARE could be useful to evaluate clinical situation of BC patients since they are independently related to short term death.

As far as concerns the clinical relevance of the studies on HDL enzymes, an increased activity and concentration of serum PON1 have been reported in post-radiotherapy BC patients [92]. Since PON1 is considered a factor of the innate immune system, these data could be of clinical relevance and could suggest an improvement in the general clinical condition of BC patients.

Some hypotheses can be advanced to explain the molecular mechanisms likely implicated in the cancer-related decrease of serum PON1 activities in BC patients. PON1 levels are genetically determined; the polymorphism *PON1*_*192*_, (with alleles termed Q and R), and *PON1*_*55*_, (with alleles L and M) are strongly associated with the enzyme activity [98, 99]. A higher incidence of BC has been observed in patients with the M allele of the L55M polymorphism [98, 99]. Other mechanisms could be involved. It is well known that some cytokines such as IL-6 and IL-8 negatively modulate PON1 synthesis. An increase of pro-inflammatory cytokines has been observed in plasma of BC patients [100, 101], therefore a downregulation of the enzyme synthesis could be suggested. In addition, PON1 is a glycoprotein, alterations of the protein glycosylation patterns could occur as previously observed in proteins during cancer progression [102].

#### Lecithin-cholesterol acyltransferase (LCAT)

Among enzymes associated to HDL, LCAT exerts a role in HDL maturation, converting free cholesterol (FC) into cholesteryl ester (CE) [88], moreover it has been suggested that LCAT can also be involved in antioxidant properties of HDL [52, 53]. A significant decrease of the plasma levels of esterified cholesterol and of the activity of LCAT was observed in BC patients with an increase of the FC/EC ratios. Moreover, a significant decrease of the levels of phosphatidylcholine, the acyl donor in the LCAT reaction, was described in patients [94]. These results suggest alterations of HDL lipid composition. A decrease of plasma LCAT activity has been confirmed by Özmen et al. in BC patients, compared to healthy subjects [103].

#### Platelet-activating factor acetylhydrolase (PAF-AH)

PAF-AH is calcium-independent phospholipase A2 activity also known as lipoprotein-associated phospholipase (PLA A_2_).PLA A_2_ catalyzes the hydrolysis of glycerophospholipids at the sn-2 position, producing bioactive lipids and signaling molecules such as lysophospholipids [104]. Lipoprotein-PLA_2_ travels mainly with plasma LDL. However about 20% is associated to the HDL surface. PAF-AH hydrolyses both PAF and oxidized phospholipids. The physiological role of PAF-AH is not completely elucidated. Either an anti-inflammatory or a pro-inflammatory effect, depending on the concentration and the availability of the potential substrates have been proposed [95, 105]. Higher activity of plasma PLA_2_ has been previously observed in BC patients with respect to healthy controls [95]. Although the factors which are at the basis of oxidative stress in BC are not elucidated, the lower activities of PON1 and the higher activities of PAF-AH could contribute to inflammation and oxidative damage of plasma lipoprotein of BC patients as previously observed in other diseases [106].

#### MicroRNAs

MicroRNAs (miRNAs) have emerged as critical regulators of cholesterol homeostasis [107] and behave as important regulators of gene expression. Since HDL particles serve as carriers of miRNAs in circulation and deliver these to cells for uptake, it has been proposed that HDL could participate to extracellular miRNA signaling. Modifications of miRNAs composition reflect in alterations of HDL functions. Recent studies suggest that miRNAs may contribute to BC and changes in the expression of miRNAs have been proposed as non-invasive biomarkers for breast cancers [108, 109]. Whether miRNAs are responsible for some alterations of HDL anti-inflammatory and antioxidant properties that could be of interest in cancer, await further testing.

#### Serum amyloid A (SAA)

Serum amyloid A (SAA) is a protein synthesized in the liver during acute phase response to inflammatory stimulus. It has been demonstrated that SAA circulates in plasma as a component of HDL [110]. The study of mechanisms which contribute to the development and progression of breast cancer has demonstrate the involvement of inflammatory pathways. In detail, the amphipathic α-helical structure in the N-terminal region of SAA plays a key role in the interactions with lipid molecules. Serum levels of SAA are considered markers of low-grade chronic inflammation and potential predictors of cancer survival in BC patients [111]. Although the content of SAA in HDL of BC patients has not been studied, an increased association of SAA at the HDL surface, may contribute to alterations of PON1 activity in response to acute inflammatory stimuli. Previous studies have shown alterations of the HDL functions and antioxidant ability due to higher levels of SAA. In detail interactions between SAA and HDL cause apoA-I displacement and these compositional changes are associated with a decreased LCAT activity and PON1 activity [111].

#### Sphingosine-1-phosphate (S1P)

Sphingosine-1-phosphate (S1P) is one of the main representatives of sphingolipids. S1P in healthy subjects is relatively more abundant in plasma than in tissues. In healthy humans, S1P is mainly present in HDL (> 55%) and to a lesser extent in other lipoproteins [112, 113]. Previous studies have suggested that HDL-bound S1P could be a useful circulating marker in cardiometabolic diseases. The involvement of HDL-bound S1P in other human diseases has been less investigated. Plasma levels of S1P positively collaborate with HDL-C and apoA-I and it has been suggested that the S1P content of HDL may modulate HDL physiological effects. A higher S1P content is correlated with HDL antioxidant properties. Alterations of levels of serum S1P have been demonstrated in patients affected by a stage IIIA breast cancer with respect to age- matched healthy volunteers [114, 115]. Furthermore, significantly higher levels of S1P have been demonstrated in breast cancer tissue. The role of HDL-S1P in cancer progression deserves of future studies.

## Effect of normal and dysfunctional HDL on breast cancer cells

**Fig. 2:**
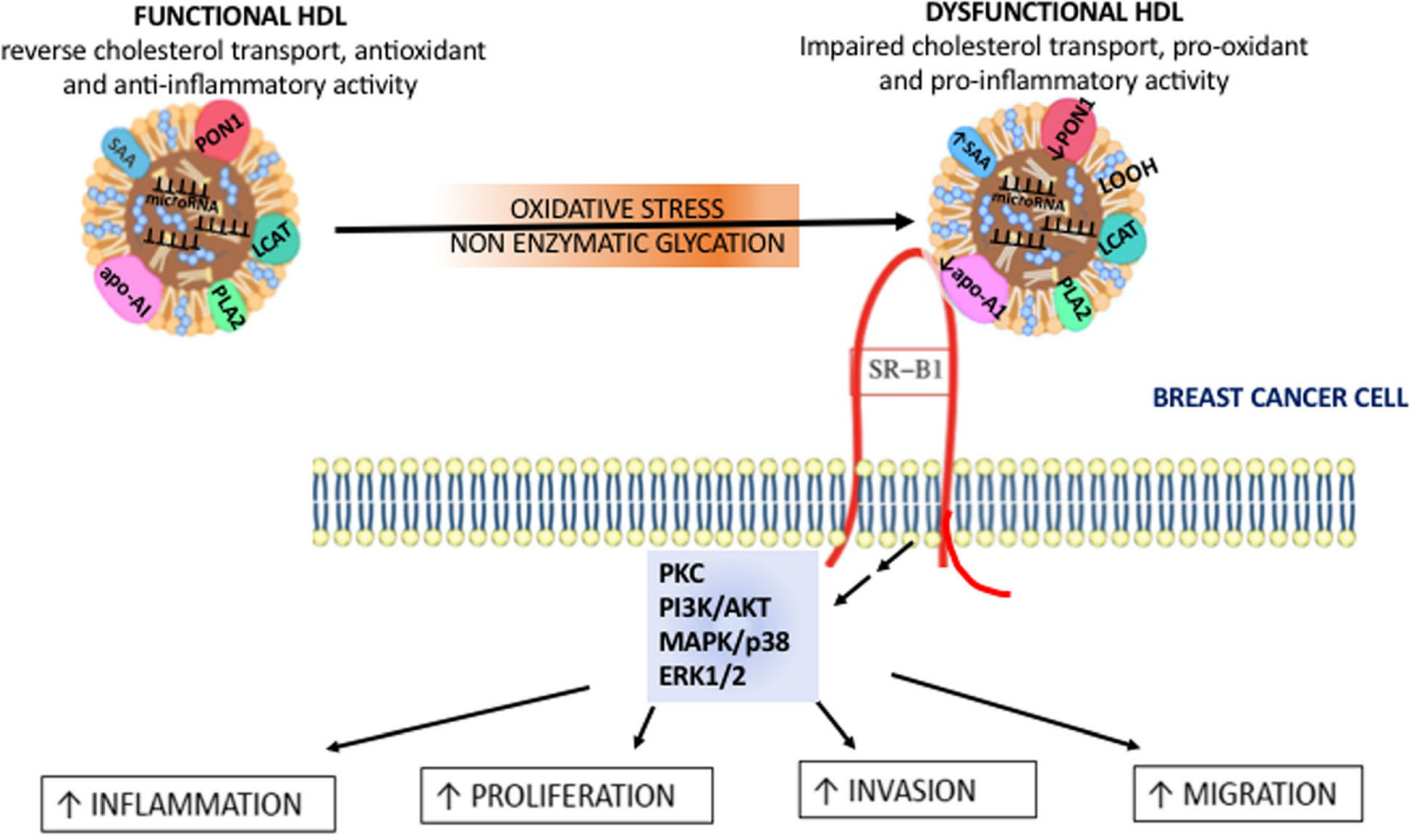
Role of high-density lipoprotein (HDL) and dysfunctional HDL on breast cancer cells. HDL undergo continuous remodeling during their transit in plasma and extravascular compartments. Oxidation and glycation of HDL can occur under metabolic conditions associated with accelerated atherosclerosis and an increased risk of coronary artery disease (CAD), such diabetes. Oxidation and glycation of HDL result in significant compositional and functional changes. Reduction of paraoxonase-1 (PON1), replacement of apo-AI with the acute phase protein serum amyloid A (SAA), reduced S1P and oxidative modifications of phospholipids (LOOH) cause impaired biological activity of HDL (functionally defective HDL or dysfunctional HDL). Interaction of HDL and dysfunctional HDL with membrane receptors, including scavenger receptor class B type (SR-BI), on breast cancer cell, leads to activation of intracellular signaling pathways resulting in an inflammatory tumor environment and induction of proliferation, migration and invasion of breast cancer cells

Two cell lines (MDA-MB-231 and MCF-7) have been mainly used to study the effects of HDL on breast cancer cells. The interest to the study of the interactions between HDL and breast cancer cells is related to their key role in lipid transport, efflux of cholesterol and products of lipid peroxidation from plasma membrane, modulation of cell multiple signaling pathways. MDA-MB-231 and MCF-7 have many phenotypic/genotypic differences. MCF7 are hormone dependent (both estrogen and progesterone receptor positive—ER and PR), while MDA-MB-231 is a poorly differentiated TNBC cell line and lacks ER and PR expression. Previous studies have shown metabolic differences. For instance the two cell types present a different response to ApoA-I and apoE expression [116]. In MCF-7 cells, both apolipoproteins decrease cholesterol transfer to the plasma membrane. On the contrary, an opposite effect was observed in MDA-MB-231 cells. Furthermore different effects of apolipoproteins on cellular proliferation and migration potential have been observed in the two cell lines [116]. Differences in the susceptibility to drugs or nutritional factors have been also described [117–119]. Table 4 summarizes the results of the studies of the effects of normal HDL and dysfunctional HDL on breast cancer cells. The effect of lipoproteins on proliferation of human breast cancer cells in culture was studied many years ago by Rotheneder et al. (1989) [120]. Marked differences were found between MCF-7 and MDA-MB-231. HDL stimulated the proliferation of both cell lines in a dose-dependent manner but MDA-MB-231 cells showed a response which realized at a higher extent. More recent studies have demonstrated that HDL modulate proliferation and migration of both BC cell models [121–123]. A role in the regulation of cellular proliferation and migration in both MCF7 and MDA-MB-231 cells is played by the interactions between HDL and the membrane scavenger receptor SR-BI and concomitant activation of the signaling pathways Akt and ERK. A lower activation of AKT and an inhibition of breast cancer cell proliferation and migration were observed in cells after knockdown of SR-BI [122]. Moreover, proliferation induced by HDL was blocked in MCF-7 cells containing a non-functional SR-BI [123]. Pan et al. [124] reported a dose-dependent effect of HDL. Normal HDL, at low concentrations, promoted MDA-MB-231 breast cancer cell capacities of adhesion and migration to HUVECs and attachment to ECM, whereas higher concentrations exerted an inhibitory effect. In vivo studies confirmed that normal HDL reduced metastasis of MCF7 cells in the lung and in the liver as compared with control. An inhibition of cell viability has been also observed in inflammatory breast cancer cell lines (SUM 149 and KPL4) incubated with HDL prior to irradiation compared to untreated cells [125]. The effects of dysfunctional HDL such as oxidized-HDL (oxHDL) or glycated HDL have also been studied due to their physio-pathological relevance. HDL oxidized in vitro stimulate cell migration, proliferation, invasion, and adhesion as demonstrated using hypochlorite-oxidized HDL [126]. The effect is likely related to the protein kinase C (PKC) pathway which modulates several cellular responses, cell proliferation and the inflammatory response. Studies in vivo have demonstrated that ox-HDL promote also metastasis to pulmonary and hepatic cells compared with normal HDL. Both glycated-HDL and ox-HDL show a higher ability to promote cell proliferation and migration of breast cancer cells involving different pathways (Akt, ERK, and mitogen-activated protein kinase (MAPK) compared with control HDL [123, 127]. Incubation of BC cells with HDL isolated from diabetic patients also promote the metastasis capacity of BC cells, increase their adhesion to HUVEC cells and attachment to the extracellular matrix compared with control HDL. The study of the molecular mechanisms has demonstrated that the effects exerted by dysfunctional HDL are due to the activation of PKC which stimulates secretion of integrins [127]. These proteins play a role in promoting breast cancer metastasis (Table 4). Figure 2 summarizes the potential roles of dysfunctional HDL on breast cancer cells. Even HDL isolated from BC patients affected by Type 2 diabetes (T2DM) promote an increased adhesion of BC cells to HUVEC and stimulate a higher expression of intercellular adhesion molecule and vascular cell adhesion molecule when compared with HDL isolated from BC patients [127]. However, in BC patients with T2DM, a lower expression of intercellular adhesion molecule and vascular cell adhesion molecules was found in tumor tissue, and it has been suggested that these alterations could be involved in the metastasis of tumor cells [127].

**Table 4:** Effect of normal and dysfunctional HDL on breast cancer cells

HDL sample	Effect on breast cancer cells	Signaling pathways involved
**Normal HDL**	Decreased metastasis of MCF7 cells in the liver compared with control animals in which HDL was not injected.	
**Hypochlorite-oxidized HDL**	Stimulation of cell proliferation, migration, invasion, and adhesion in vitro Promotion of breast cancer cell pulmonary and hepatic metastasis compared with normal HDL in vivo.	Protein kinase C (PKC) pathway
**Glycated HDL** **Copper-oxidized HDL**	Stimulation of cell proliferation, migration, invasion, and adhesion in vitro increased capacity of adhesion to human umbilical vein endothelial cells (HUVECs) compared with normal HDL	Protein kinase C (PKC) pathway
**HDL isolated from T2DM patients**	Promoted cell proliferation, migration, and invasion of breast cancer cells Promoted the metastasis capacity of breast cancer cells in vivo Increased capacity of adhesion to human umbilical vein endothelial cells (HUVECs) compared with normal HDL	Activation of Akt, ERK, and p38 mitogen-activated protein kinase (MAPK) pathways
**HDL isolated from breast cancer patients complicated with T2DM**	Promoted breast cancer cell adhesion to HUVECs and stimulated higher intercellular adhesion molecule 1 (ICAM-1) and vascular cell adhesion molecule 1 (VCAM-1)	Activation of PKC pathway ERK and p38 MAPK pathways

## Strengths and limitations of the review

The relationship between levels of HDL-C and apoA-I and breast cancer have been widely investigated and reviewed. In our review we focused our attention on the relationship between breast cancer and markers of oxidative stress of plasma lipids. We reviewed literature data on circulating levels of products of lipoperoxidation. Moreover, we summarized data on modifications of the activities of HDL enzymes in BC patients. These topics have not been previously reviewed. A limit of the review is related to the few studies which have been included. Infact, markers of lipid peroxidation and activities of HDL-enzymes have been less investigated with respect to HDL-C levels.

## Conclusions

Previous clinical studies indicate a negative relationship between levels of HDL-C and breast cancer risk. Contrasting results have been reported by other authors. However, several studies have shown that HDL-C does not reflect accurately HDL physiological roles. Higher plasma levels of markers of lipid peroxidation and alterations of activities of enzymes which exert key roles in HDL properties are described in breast cancer patients. HDL functionality is affected by its apolipoprotein composition and activity of enzymes. The lower LCAT activity suggest alterations of cholesterol metabolism. Moreover, the lower activities of enzymes involved in the antioxidant properties of HDL such as PON1 and PLA_2_ suggests this key role of HDL could be impaired in BC patients. The higher plasma PLA_2_ activity detected in patients with breast cancer can contribute to production of potent bioactive lipids such as lysophospholipids and oxidized fatty acids. All these data suggest dysfunctional HDL in breast cancer patients. Dysfunctional HDL exert a lower protective affect against lipid peroxidation and induce proliferation and migration of breast cancer cells. Further studies are necessary to demonstrate the molecular mechanisms which could contribute to activation of inflammatory pathways and an increased risk of breast cancer. We suggest that the study of HDL functions using in vitro assays could be useful to evaluate alterations of HDL and help advise patients about lifestyle modifications, nutritional factors and/or drugs that modulate HDL levels and functions.

## Data Availability

Not applicable.

## Abbreviations

apoA-I: Apolipoprotein A-I
ARE: Arylesterase activity
BC: Breast cancer
CE: Cholesteryl ester
CEOOH: Cholesterol esterified hydroperoxides
CM: chylomicron
CT: cholestane-3β,5α,6β-triol
27-HC: 27-Hydroxycholesterol
5,6-EC: 5,6 Epoxycholesterol
EGFR: Epidermal growth factor receptor
FC: Free cholesterol
HDL: High density lipoproteins
ICAM-1: Intercellular adhesion molecule 1
LCAT: Lecithin cholesterol acyltransferase
LDL: Low density lipoprotein
LOOH: Lipid hydroperoxides
MDA: Malondialdehyde
miRNAs: MicroRNAs
OCDO: 6-oxo-cholestan-diol
oxLDL: Oxidized LDL
oxHDL: Oxidized HDL
OS: Oxysterols
PAF-AH: Platelet activating factor acetyl hydrolase
PKC: Protein kinase C
PLA2: Phospholipase A2
PON1: Paraoxonase 1
S1P: Sphingosine-1-phosphate
SAA: Serum amyloid A
SR-BI: Scavenger Receptor class B type I
T2DM: Type 2 diabetes mellitus
TNBC: Triple negative breast cancer
VCAM-1: Vascular cell adhesion molecule 1
